# Small RNA mediated repression of subtilisin production in *Bacillus licheniformis*

**DOI:** 10.1038/s41598-017-05628-y

**Published:** 2017-07-18

**Authors:** Robert Hertel, Sandra Meyerjürgens, Birgit Voigt, Heiko Liesegang, Sonja Volland

**Affiliations:** 1 Department of Genomic and Applied Microbiology & Goettingen Genomics Laboratory, Institute of Microbiology and Genetics, University of Goettingen, Goettingen, Germany; 2 Institute for Microbiology, Ernst-Moritz-Arndt University, Greifswald, Germany; 3 Research Institute of Leather and Plastic Sheeting gGmbH, Freiberg, Germany

## Abstract

The species *Bacillus licheniformis* includes important strains that are used in industrial production processes. Currently the physiological model used to adapt these processes is based on the closely related model organism *B*. *subtilis*. In this study we found that both organisms reveal significant differences in the regulation of subtilisin, their main natural protease and a product of industrial fermentation processes. We identified and characterized a novel antisense sRNA AprAs, which represents an RNA based repressor of *apr*, the gene encoding for the industrial relevant subtilisin protease. Reduction of the AprAs level leads to an enhanced proteolytic activity and an increase of Apr protein expression in the mutant strain. A vector based complementation of the AprAs deficient mutant confirmed this effect and demonstrated the necessity of *cis* transcription for full efficiency. A comparative analysis of the corresponding genome loci from *B*. *licheniformis* and *B*. *subtilis* revealed the absence of an *aprAs* promoter in *B*. *subtilis* and indicates that AprAs is a *B*. *licheniformis* species specific phenomenon. The discovery of AprAs is of great biotechnological interest since subtilisin Carlsberg is one of the main products of industrial fermentation by *B*. *licheniformis*.

## Introduction

*Bacillus licheniformis* was originally isolated and described by Weigman in 1898^1^. The species is a member of the *B*. *subtilis* species complex^2^ and exhibits a saprophytic life style on organic material^3, 4^. The lifestyle results in three properties which promote *B*. *licheniformis* as platform for productive fermentations. (I) *Bacilli* can use a broad spectrum of plant derived C- and N-sources^5, 6^. (II) Since the organisms live on organic matter they are evolved for high concentrations of nutrient supply which enables the them to reach high cell densities in fermentations^7^. Finally, (III) they secrete high amounts of many efficient bio-polymer hydrolases^8^ like lichenases^9^, lipases^10^, thermostable alpha-amylases^11^, and many more^12–20^. Exoproteases of the subtilisin Carlsberg family are an industrial fermentation product of *B*. *licheniformis* and *B*. *subtilis* strains with an annual production rate of about 500 t^21^. Subtilisin is highly thermostable, active under alkaline conditions (pH 10–11) and, due to its low substrate specificity, an important additive for household detergents^21, 22^.

In 2004 the type strain *B*. *licheniformis* DSM13 (also ATCC14580) was sequenced by Rey *et al*. (Davis, USA)^23^ and Veith *et al*. (Göttingen, Germany)^24^ revealing a plasmid free 4.2 Mbp genome. Almost a decade later, the RNA based regulatory network of *B*. *licheniformis* DSM13 was analysed by RNA-Seq using samples from different stages of an industrial fermentation^25^.The transcriptome data revealed a considerable number of protein encoding RNAs and also 3,314 non-coding RNAs, divided in 2853 mRNA-bound and 461 small RNAs. In general, small RNAs (sRNA) are versatile regulators of gene expression, which can facilitate their function *in cis* or *trans*, controlling e.g. mRNA stability, degradation, termination and translation^26, 27^. Three prominent classes of sRNAs have been described: (i) structured sRNAs with limited complementarity to their targets, (ii) sRNAs that bind regulatory proteins and (iii) antisense RNAs synthesized from the strand complementary to their target mRNA^28^.

In the transcriptome of *B*. *licheniformis* an antisense RNA element on the 3′ end of the *apr* gene, encoding the subtilisin protease, gained considerable attention, since the samples were taken from a subtilisin fermentation^25^. Due to its association with *apr*, we called the RNA element AprAs. Its active region has a length of 144 nucleotides and shows complete sequence identity to the antisense 3′ coding region of the *apr* gene. The RNA-Seq data from Wiegand *et al*.^25^ also indicated that the small RNA is transcribed by its own promoter. Its transcription rate exceeds the transcription rate of the *apr* mRNA by three orders of magnitude.

For the closely related organism *B*. *subtilis* no comparable antisense RNA associated with the 3′-end of *aprE* was described, although extensive transcriptome analyses were performed by Rasmussen *et al*. in 2009^29^ and Nicolas *et al*. in 2012^30^. The subtilisin gene *aprE* in *B*. *subtilis* was found to be regulated by the interacting global transcriptional regulators CodY and ScoC as well as by AbrB^31^. Barbieri *et al*.^31^ could also show that the regulatory network in *B*. *subtilis* results in a repression of the *aprE* gene within the exponential growth phase and an exclusive expression of AprE within the stationary phase. The genome of *B*. *licheniformis* DSM13 also exhibits orthologues of *aprE* regulators^23, 24^ and the transcriptome analysis of *B*. *licheniformis* DSM13^25^ showed a corresponding expression pattern for *apr*. However, the association of the *apr* gene with a highly expressed small antisense RNA indicates an additional regulatory layer in *B*. *licheniformis*.

The here presented work focuses on the impact of the newly discovered antisense RNA AprAs. By inactivation of the *aprAs* promoter and vector encoded transcription of AprAs we could show its regulatory effect on proteolytic activity and Apr expression.

## Results

### Construction and characterization of an AprAs deficient mutant

**Figure 1:**
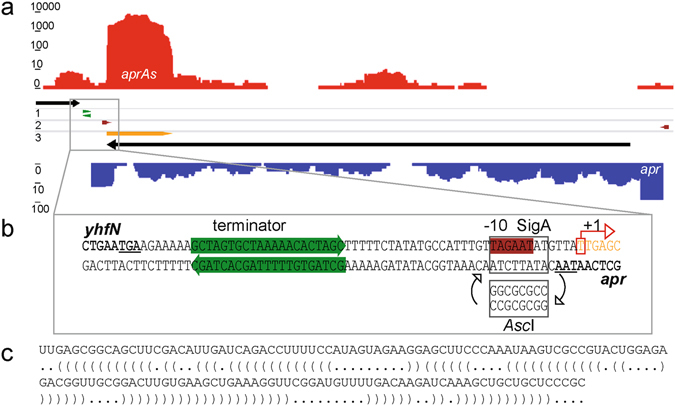
AprAs transcription profile, promoter region and sequence analysis. (**a**) Transcription profile of AprAs in the early stage of an industrial subtilisin production^25^. Transcriptome data were visualised in a logarithmic scale using TraV^56^. The red graph represents the transcription activity of *aprAs* and the blue of *apr*. The black arrow on the complementary strand represents the coding region of *apr* and the yellow arrow of *aprAs*. The dark red arrows represent the identified promoters and green arrows predicted transcription terminators. (**b**) Promoter region of *aprAs*. The −10 box (TAGAAT) of the potential SigA promoter is highlighted in red and the exchanged sequence (*Asc*I pattern) is given. Predicted transcription terminators are framed with green arrows. Protein coding sequences are shown in black bold letters and the stop codons are underlined. The *aprAs* coding sequence is given in orange letters and the experimentally determined transcription start site of AprAs^25^ is framed in red. (**c**) AprAs sequence. The 144 base large AprAs sequence was analyzed via the RNAfold WebServer, using “The Vienna RNA Websuite”^55^. The calculation of secondary structures was possible and the optimal structure with a minimum free energy of −51.9 kcal/mol is shown in dot-bracket notation.

**Figure 2:**
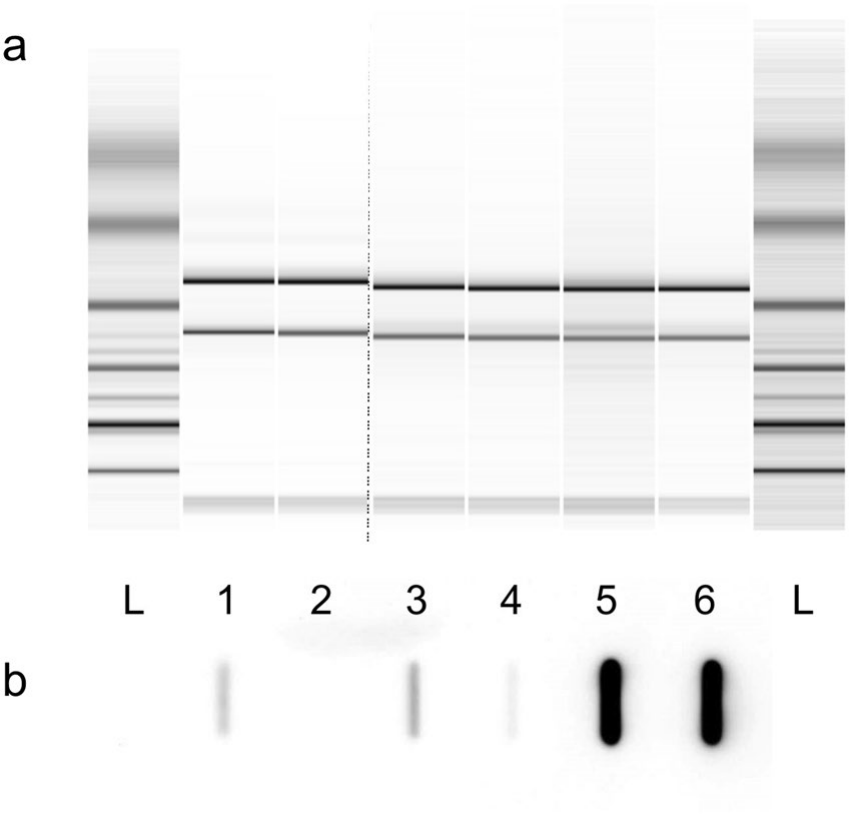
Slot blot analysis of AprAs transcription in *B*. *licheniformis* MW3 and *B*. *licheniformis* MW3 mutant strains. (**a**) The quality of the total RNA of *B*. *licheniformis* MW3 (lane 1), *B*. *licheniformis* MW3 AprAs^−^ (lane 2), *B*. *licheniformis* MW3 pV2 (lane 3), *B*. *licheniformis* MW3 AprAs^−^ pV2 (lane 4), *B*. *licheniformis* MW3 pV2::*aprAs* (lane 5) and *B*. *licheniformis* MW3 AprAs^−^ pV2::*aprAs* (lane 6) was determined using an Agilent Bioanalyzer. Lane 1 and 2 were analysed on a separate chip and the respective ladders (L) are shown. (**b**) The AprAs transcript was detected with an AprAs specific probe in *B*. *licheniformis* MW3 total RNA. *B*. *licheniformis* MW3 (lane 1) and *B*. *licheniformis* MW3 pV2 (lane 3) show an AprAs specific signal, whereas *B*. *licheniformis* MW3 AprAs^−^ and *B*. *licheniformis* MW3 AprAs^−^ pV2 (lane 4) possess no or only a very faint AprAs specific signal. *B*. *licheniformis* MW3 pV2::*aprAs* (lane 5) and *B*. *licheniformis* MW3 AprAs- pV2::*aprAs* (lane 6) show a strong AprAs signal dependent on the AprAs transcription from vector pV2::*aprAs*.

To investigate the impact of the 144 base antisense sRNA AprAs (Bli_r0872 in *Wiegand et al*.^25^), an AprAs deficient *B*. *licheniformis* MW3 mutant was generated. A direct deletion of *aprAs* was not possible due to its overlap with the *apr* gene on the complementary DNA strand (see Fig. 1a). However, the previous RNA-Seq experiment^25^ also included differential RNA-Seq (dRNA-Seq, see Sharma *et al*.^32^) and thus, the transcription start site of *aprAs* and a corresponding promoter pattern could be identified. The SigA pattern was located on the positive DNA strand between the stop codon of the *apr* coding region and its transcription terminator (see Fig. 1b). In order to prevent the transcription of AprAs, the AT-rich region of the −10 promoter box was replaced with the GC-rich recognition pattern of the *Asc*I endonuclease. For easy detection of the desired mutation, a 2.6 kbp PCR product was amplified from the respective genomic region and digested with the endonuclease *Asc*I. The mutant strain *B*. *licheniformis* MW3 AprAs^−^ exhibited the expected 1.3 kbp DNA band consisting of the degradation products of the 2.6 kbp PCR product (see Supplementary Figure S1). A slot blot experiment confirmed that no transcriptional activity of the *aprAs* gene was detectable in the mutant strain compared to the original strain *B*. *licheniformis* MW3 (see Fig. 2).

**Figure 3:**
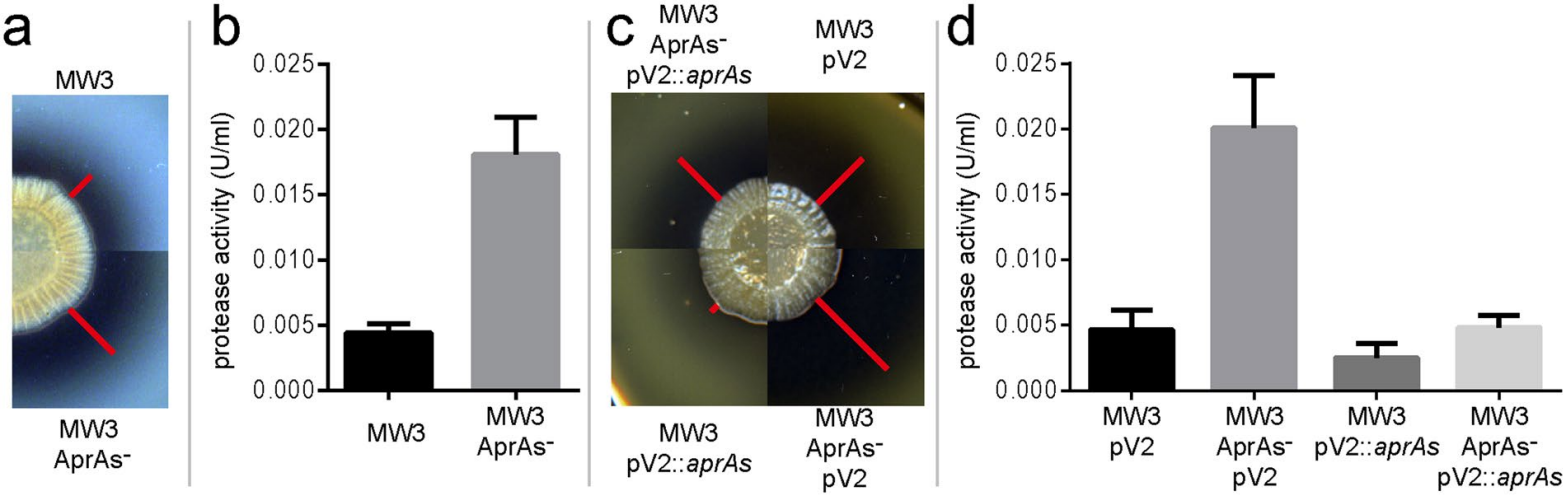
Determination of exoprotease activity by qualitative and quantitative measurement in *B*. *licheniformis* MW3 and mutant strains. For qualitative determination of exoprotease activity (**a**,**c**), cultures were adjusted to OD_600_ of 0.1. Of each culture 3 µl were dropped on a M9 skim milk agar plate and incubated for 5 days at 37 °C. Exoprotease activity led to the digestion of milk protein resulting in a surrounding halo. The size of the respective halos is indicated with a red bar. For quantitative determination of exoprotease activity (**b**,**d**), all cultures were inoculated to an OD_600_ of 0,1 in liquid M9 skim milk medium and incubated for 24 h under intensive shaking. The exoprotease activity was determined using the culture supernatant. (**a**) *B*. *licheniformis* MW3 AprAs^−^ reveals an increased exoprotease activity visible through a stronger halo formation compared to MW3. (**b**) The four fold increased exoprotease activity of *B*. *licheniformis* MW3 AprAs^−^ confirms the exoprotease phenotype observed in Fig. 3a. (**c**) *B*. *licheniformis* MW3 pV2::*aprAs* shows the smallest halo formation. The halo of the complemented *B*. *licheniformis* MW3 AprAs^−^ pV2::*aprAs* is comparable to MW3 pV2. *B*. *licheniformis* MW3 AprAs^−^ pV2 shows the strongest proteolytic activity indicated by the largest halo formation. The ectopic transcription of *aprAs* results in a reduction of exoprotease activity in both MW3 and mutant AprAs^−^. (**d**) The protease activity of the complemented strain *B*. *licheniformis* MW3 AprAs^−^ pV2::*aprAs* is strongly decreased compared to *B*. *licheniformis* MW3 AprAs^−^ and comparable to *B*. *licheniformis* MW3 pV2. *B*. *licheniformis* MW3 pV2::*aprAs* shows an approximately 50% reduced exoprotease activity.

To investigate the impact of AprAs on the protease activity of *B*. *licheniformis*, the AprAs^−^ mutant and the original strain MW3 were grown on M9 skim milk agar plates. *B*. *licheniformis* MW3 AprAs^−^ exhibited increased halo formation, indicative for a stronger proteolytic activity compared to strain MW3 (Fig. 3a). These qualitative results were confirmed by quantitative non-specific exoprotease assays using 24 h M9 skim milk liquid cultures. Here, the AprAs deficient strain *B*. *licheniformis* MW3 AprAs^−^ showed an approximately four fold increased exoprotease activity compared to *B*. *licheniformis* MW3 (see Fig. 3b).

### Verification of AprAs phenotype

*B*. *licheniformis* MW3 AprAs^−^ was complemented with a vector encoded *aprAs* gene to restore the original phenotype and confirm that the observed exoprotease phenotype of *B*. *licheniformis* MW3 AprAs^−^ is based on the inactivation of AprAs transcription. The *aprAs* gene with its native promoter was cloned into the *Bacillus*/*E*. *coli* shuttle vector pV2^33^, creating pV2::*aprAs*. The vector was introduced into *B*. *licheniformis* MW3 AprAs^−^ and the original strain *B*. *licheniformis* MW3. The vector pV2 was used as a control. The slot blot analysis (Fig. 2) confirmed the re-establishment of AprAs transcription in *B*. *licheniformis* MW3 AprAs^−^ pV2::*aprAs*. *B*. *licheniformis* MW3 pV2 exhibited AprAs transcription due to the chromosomal *aprAs* gene. *B*. *licheniformis* MW3 pV2::*aprAs* showed an increased AprAs transcription due to expression from the chromosomal *aprAs* gene in addition to the multi-copy vector encoded *aprAs* gene.

The qualitative evaluation of the protease activity on M9 skim milk agar plates (Fig. 3c) showed that the complemented mutant strain *B*. *licheniformis* MW3 AprAs^−^ pV2::*aprAs* could re-establish the “wild type” phenotype. Its halo formation was comparable to *B*. *licheniformis* MW3 pV2 and reduced in comparison to *B*. *licheniformis* MW3 AprAs^−^. These qualitative results were confirmed by the quantitative exoprotease activity evaluation (Fig. 3d), determined in the supernatant of 4 ml culture in liquid M9 skim milk media. The reduction of AprAs transcription in *B*. *licheniformis* MW3 AprAs^−^ resulted once more in an approximately 4 times increased exoprotease activity. This effect was reversed by the ectopic AprAs transcription in *B*. *licheniformis* MW3 AprAs^−^ pV2::*aprAs*. The *in trans* overexpression of AprAs in the original strain MW3 resulted in an additional reduction of exoprotease activity of approximately 50% (see *B*. *licheniformis* MW3 pV2::*aprAs*).

### Impact of AprAs on Apr expression

**Figure 4:**
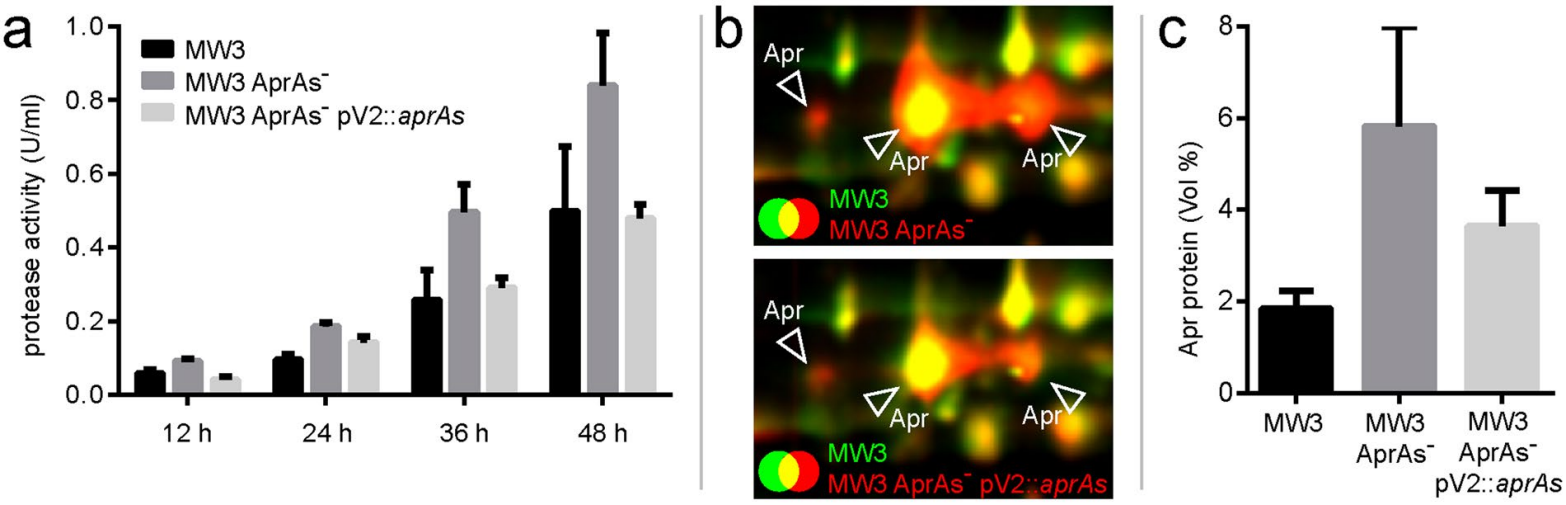
Protease activity and Apr protein expression in *B*. *licheniformis* MW3, MW3 AprAs^−^ and MW3 AprAs^−^ pV2::*aprAs*. The strains were grown in 400 ml M9 skim milk medium for 48 h. (**a**) The cell culture supernatants of samples from time points 12 h, 24 h, 36 h and 48 h were used for quantitative exoprotease activity determination. The absolute value of protease activity is shown as the average of three independent experiments, each with triplicate measurements. The exoprotease activity increased from 12 h to 48 h and is clearly stronger in *B*. *licheniformis* MW3 AprAs^−^ in all experiments compared to *B*. *licheniformis* MW3 and MW3 AprAs^−^ pV2::*aprAs*. The error bars display the standard deviation and the colour legend is shown in the figure. (**b**) Extracellular proteins were isolated from supernatants of 400 ml M9 skim milk cultures after 48 h of growth. The protein fractions were separated by 2D-gelelectrophoresis. A gel image of *B*. *licheniformis* MW3 (green) was overlaid with the respective image of MW3 AprAs^−^. The volume of the three identified Apr protein spots is strongly increased in *B*. *licheniformis* MW3 AprAs^−^ (red) compared to the original strain *B*. *licheniformis* MW3 (green). In addition, the gel image of *B*. *licheniformis* MW3 (green) was overlaid with the respective image of MW3 AprAs^−^ pV2::*aprAs* (red). After vector complementation, the effect of the mutation is reversed. *B*. *licheniformis* MW3 AprAs^−^ pV2::*aprAs* shows only a slight increase in Apr protein expression compared to *B*. *licheniformis* MW3. (**c**) To quantify the Apr expression the spot volumes (in percentage of the whole protein spot volume) were calculated. The average of the normalized spot volumes of three replicates is shown and the standard deviation is given. The Apr protein expression in the extracellular proteome of *B*. *licheniformis* MW3 AprAs^−^ is approximately three times increased compared to the original strain *B*. *licheniformis* MW3. The Apr expression of *B*. *licheniformis* MW3 AprAs^−^ pV2::*aprAs* is reduced compared to the precursor strain *B*. *licheniformis* MW3 AprAs^−^, but still higher than in *B*. *licheniformis* MW3.

In order to investigate the correlation between the AprAs level and the expression of the protease Apr, the extracellular proteomes of the original strain MW3, the AprAs^−^ mutant and the complemented strain were analysed by 2D-gelelectrophoresis and MALDI-TOF-MS/MS^34^. All strains were grown in 400 ml liquid M9 skim milk medium and supernatants were harvested after 12 h, 24 h, 36 h and 48 h. The quantitative exoprotease assay was performed using the supernatants of three experiments (Fig. 4a). The overall proteolytic activity of the strains increased constantly from 12 h to 48 h. The AprAs deficient mutant strain *B*. *licheniformis* MW3 AprAs^−^ showed the highest activity levels with approximately 0.8 U/ml after 48 h. The initial strain *B*. *licheniformis* MW3 and the vector complemented strain MW3 AprAs^−^ pV2::*aprAs* showed both lower activity levels with the highest level of approximately 0.5 U/ml. The samples with the highest activity levels (48 h) were analysed in triplicates by 2D-gelelectrophoresis. Three Apr spots were identified in the supernatants of the cultures. Figure 4b exemplarily shows the dual channel images of 2D-gels from the strain *B*. *licheniformis* MW3 compared to the mutant strains *B*. *licheniformis* MW3 AprAs^−^ and *B*. *licheniformis* MW3 AprAs^−^ pV2::*aprAs* respectively. The first image demonstrates a strong increase of Apr protein expression in the extracellular proteome of the AprAs^−^ mutant compared to MW3. This effect is reversed after vector complementation of the mutant strain: *B*. *licheniformis* MW3 AprAs- pV2::*aprAs* shows only a slight increase in Apr expression compared to the original strain *B*. *licheniformis* MW3. To quantify the differences in Apr protein expression, the spot volumes of the Apr protein spots of all three replicates were compared. Figure 4c shows that the Apr expression in the extracellular proteome of *B*. *licheniformis* MW3 AprAs^−^ was three times increased compared to the original strain *B*. *licheniformis* MW3. After vector complementation of the AprAs^−^ mutant, a reduction of the Apr protein level was visible (see *B*. *licheniformis* MW3 AprAs^−^ pV2::*aprAs*), but was still two times increased in comparison to the original strain MW3.

The complete dual channel images of the compared strains (Supplementary Figure S2) showed no differential expression for protein spots of other known extracellular *B*. *licheniformis* proteases, such as the metalloprotease Mpr and the minor extracellular protease Vpr (Vpr_1–Vpr_6), as well as the extracellular bacillopeptidase Bpr (Bpr_1, Bpr_2).

### Sequence comparison of the *AprAs* promoter region

**Figure 5:**
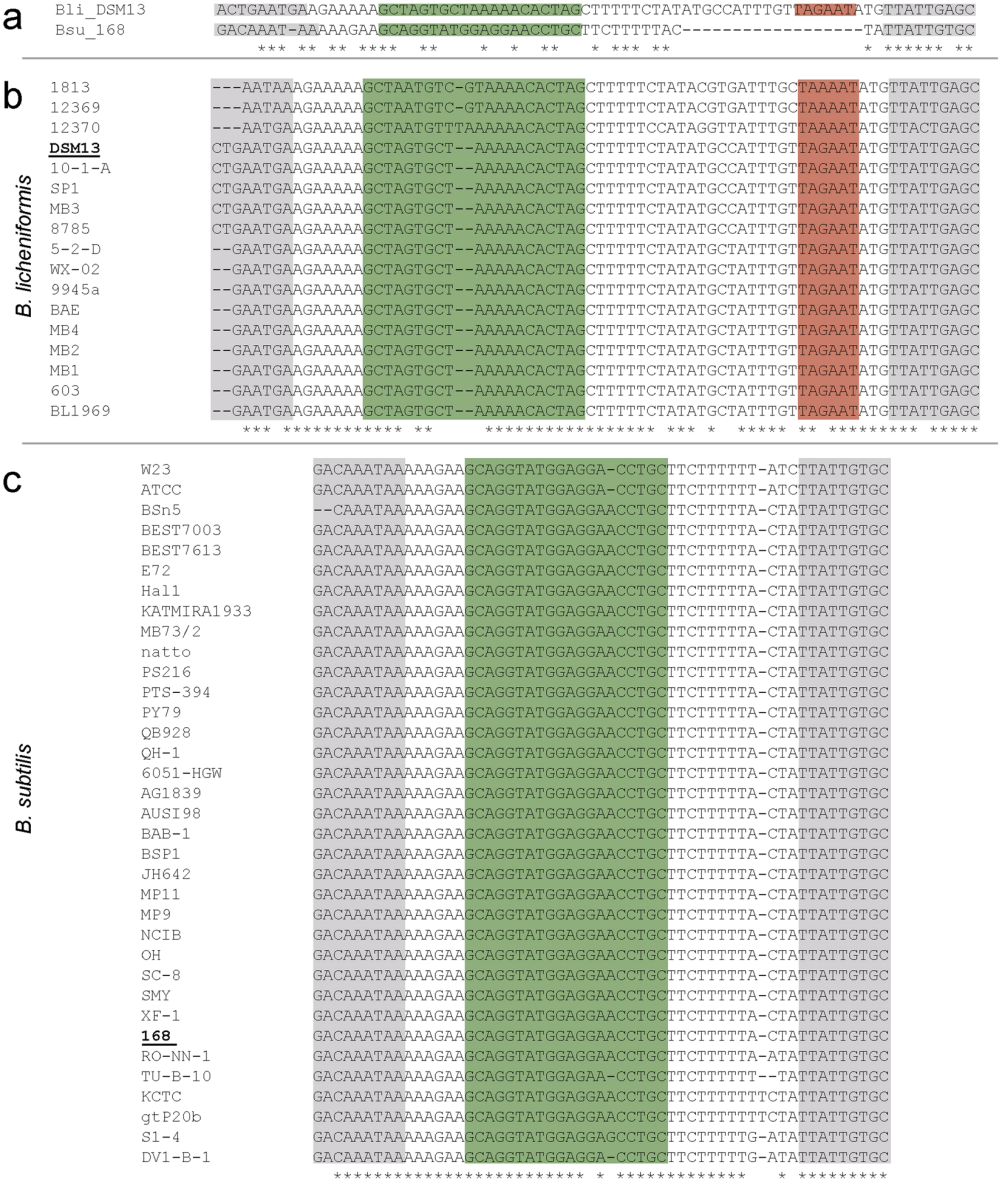
Alignment of AprAs promoter sequences from *B*. *licheniformis* and *B*. *subtilis strains*. Protein coding sequences are marked in grey. Transcription terminator sequences are marked in green and the promoter regions (−10 box) in red. The alignment was performed using MUSCLE^53^. (**a**) Sequence alignment of *B*. *licheniformis* DSM13 (NC_006270) and *B*. *subtilis* 168 (NC_000964). *B*. *subtilis* 168 lacks the −10 box upstream of the *aprAs* gene of *B*. *licheniformis* DSM13. (**b**) Sequence alignment of 17 *B*. *licheniformis* strains. The AprAs −10 box is present in all strains. (**c**) Sequence alignment of 35 *B*. *subtilis* strains. The AprAs −10 box is absent in all strains.

Orthologues of the subtilisin gene are found in *B*. *licheniformis* and *B*. *subtilis* strains. Yet, to our knowledge no orthologous *aprAs* genes associated with *apr* genes were described in the transcriptome analyses of *B*. *subtilis* 168 from Rasmussen *et al*. in 2009^29^ and Nicolas *et al*. in 2012^30^. To find out if *aprAs* and especially its promoter region are conserved, we performed a comparative sequence analysis. The comparison (Fig. 5) comprised the intergenic region between *yfhN* and *apr* respectively *aprE*, with a special focus on the −10 promoter box (green coloration), which was experimentally verified to be responsible for the transcription of AprAs in *B*. *licheniformis* DSM13 in the present investigation (see also Fig. 1). The comparison of *B*. *licheniformis* DSM13 and *B*. *subtilis* 168 showed that the *aprAs* promoter box (green coloration) is missing in *B*. *subtilis* 168. (Fig. 5a). To clarify if AprAs is a strain or species specific phenomenon, we also aligned the concerning regions of 17 *B*. *licheniformis* strains (see Fig. 5b) and 35 *B*. *subtilis* strains (see Fig. 5c). The sequence comparison showed that the *aprAs* promoter is present in all investigated *B*. *licheniformis* strains but not in the compared *B*. *subtilis* strains.

## Discussion

Our results clearly reveal the small RNA AprAs as a repressor of the protease Apr. The protease activity measurements also demonstrated the necessity of *cis* transcription of AprAs in relation to *apr* for highly efficient repression. The observed protease phenotypes of the initial strain and the complemented strain *B*. *licheniformis* MW3 AprAs^−^ pV2::*aprAs* were on the same level, showing that the transcription of one *aprAs* gene *in cis* amounts to approximately 50 copies^33, 35^ *in trans*. The combination of the single *cis* encoded *aprAs* gene and the multi-copy vector in *B*. *licheniformis* MW3 pV2::*aprAs* reduced the exoprotease activity even 50% below the initial level (Fig. 3c,d).

The exoproteome analysis (Fig. 4) showed the direct correlation between the observed protease related phenotype and the expression of the protease Apr. The comparison of MW3 and mutant proteomes revealed Apr as the only differentially expressed extracellular protease (Supplementary Figure S2). Protein spots of other known extracellular proteases^36, 37^ did not change in relation to the AprAs level. Thus, the observed protease phenotype was facilitated by the negative regulation of AprAs on Apr expression. Although the Apr protein expression levels in *B*. *licheniformis* MW3 and MW3 AprAs^−^ pV2::*aprAs* did not exactly correspond to the levels of the proteolytic activity, both analyses clearly indicate AprAs to be a repressor of Apr expression. Antisense RNA regulation on protein expression in *Bacilli* has been described before^27^. However, this is, to our knowledge, the first time that an RNA repressor of a biotechnologically relevant product has been reported.

A requirement of the proteome analysis was the scale up of our experiment from 4 ml test tubes to 400 ml flask culture. Interestingly, after scaling up, we observed only a doubling of the proteolytic activity (Fig. 4a) in contrast to the four fold increased activity observed in the 4 ml volume experiment (Fig. 3b and d). Most likely, these differences result from the adaption of the experimental design, which can have an impact on productivity^38^, but might also reflect additional regulatory layers. Investigations on the orthologous gene *aprE* from *B*. *subtilis* 168 revealed a complex regulatory network of the subtilisin protease including, as mentioned before, regulators such as ScoC, SinR, AbrB, DegU and further associated proteins^31, 39^. The genome of *B*. *licheniformis* DSM13^23, 24^ encodes orthologues of these regulators, thus a regulatory network of similar complexity can be assumed.

In case of AprAs, no orthologous sRNA was identified in *B*. *subtilis* 168^29, 30^. Our sequence comparison of the promoter box of *aprAs* showed its presence in all investigated *B*. *licheniformis* strains and its absence in all investigated *B*. *subtilis* strains. Therefore we assume that AprAs is a species specific sRNA and thus, our results demonstrate a clear difference on subtilisin protease regulation between *B*. *licheniformis* and *B*. *subtilis*. It remains unclear if a similar regulatory layer on the subtilisin protease exists in other *B*. *subtilis* species complex members or if it is unique for *B*. *licheniformis*.

Transcriptome analysis in *B*. *subtilis* and *S*. *aureus* revealed that antisense activities can arise from inefficient termination of the sense transcription or from spurious initiation by alternative sigma factors^30, 40^. However, the strong transcriptional activity and the distinct transcription start site as well as the presence of a conserved SigA promoter pattern (Figs 1 and 5) support the hypothesis that AprAs is a real non-coding RNA and therefore a relevant regulatory layer of Apr expression.

The regulation by AprAs could involve a pairing of the small RNA and its mRNA target leading to a guided degradation, as has been discussed for antisense regulators in Thomason and Storz^41^ and in Desnoyers *et al*.^42^. The transcription of AprAs is three orders of magnitude stronger than the transcription of its target RNA^25^, which resembles viral toxin/antitoxin type I systems^43^, where the sense/antisense duplex is guided to degradation by RNaseIII^44^. Apparently, a quantitative titration of the target mRNA by the small RNA is involved in the regulatory mechanism. This is supported by the observation that in Real-time PCR analysis the *apr* mRNA level varies in relation to the AprAs level in the investigated mutant strains (Supplementary Figure S3). However, since a *cis* encoded *aprAs* is more effective than multiple vector encoded gene copies, an additional regulatory layer is possible. Figure 1c shows that AprAs might form a secondary structure which could delay the interaction of the sRNA and target mRNA. Hence, the transcription of AprAs close to its target mRNA could be an advantage that may explain the differences in efficiency between *cis* and vector encoded AprAs.

It has been shown that the production of subtilisin in *B*. *subtilis* starts at the beginning of the stationary phase^31^. In contrast, Wiegand *et al*.^25^ observed a delay between the beginning of the transcriptional activity of the *apr* gene at the end of the exponential growth phase and the actual occurrence of the Apr protease spot in the proteome of *B*. *licheniformis*. It is tempting to assume that an AprAs guided degradation of the *apr* mRNA may be responsible for this delay between transcription and expression of the *apr* gene. However, life time evaluations of small RNAs in a complex industrial high density medium are heavily challenged by the background RNase activity within the fermentation process.

The production of subtilisin proteases in *Bacilli* is of great biotechnological relevance since these production systems are extensively used to produce the main enzymatic components of household detergents^8^. Thus, a fourfold increase of the subtilisin activity, as has been achieved with the *B*. *licheniformis* wild type subtilisin gene in the AprAs^−^ mutant, is very promising. Although *apr* orthologues have been identified in most genomes of members of the *B*. *subtilis* species complex, the presence of AprAs-like RNA repressors seems less ubiquitous. AprAs was not identified in *B*. *subtilis* 168^29, 30^ or in the 35 investigated *B*. *subtilis* genomes (Fig. 5). However, the possibility to achieve an increased subtilisin production by an AprAs^−^ mutation in *B*. *licheniformis* has been demonstrated in our study and should be evaluated for *B*. *licheniformis* based fermentations of subtilisin-like enzymes.

## Conclusion and outlook

The newly identified antisense sRNA AprAs was shown to be a negative regulator of the biotechnologically important subtilisin family protease Apr in the type strain *Bacillus licheniformis* DSM13. The prevention of AprAs transcription led to an up to 4-fold increase of exoprotease activity as a result of the increased Apr protein expression. This effect was reversed by ectopic overexpression of AprAs and correspondingly, the native AprAs transcription in addition to the ectopic AprAs expression led to a decrease of proteolytic activity, even below the natural level. AprAs represents a new regulatory feature of Apr expression in *B*. *licheniformis* which is not present in the model organism and its close relative *B*. *subtilis* 168. Sequence comparison revealed its presence in all investigated *B*. *licheniformis* strains and its absence in all compared *B*. *subtilis* strains, leading to the conclusion of a species-specific feature. Further analyses should focus on revealing the detailed mechanism of AprAs regulation and the search for AprAs homologues in other *Bacilli* and industrially used *apr* gene loci.

## Material and Methods

### Bacterial strains and culture conditions

The bacterial strains used in this study are listed in Table 1. If not stated otherwise, the strains were grown in NB medium at 37 °C. M9 medium was prepared as described by Sambrook and Russell^45^. 1 L of M9 medium was additionally supplemented with 100 µl of SL-8 trace element solution as described by Atlas^46^ and 100 µl vitamin solution. The vitamin solution contained per litre 50 mg pantothenic acid, 50 mg riboflavin, 10 mg pyridoxamine-HCl, 20 mg biotin, 20 mg folic acid, 25 mg niacin, 25 mg nicotinamide, 50 mg α-aminobenzoic acid, 50 mg thiamine-HCl and 50 mg cobalamine dissolved in H_2_O. M9 medium was supplemented with a final concentration of 0.1% (w/v) sterile skim milk to generate M9 skim milk medium. For medium solidification agar with a final concentration of 1.5% (w/v) was added prior to sterilisation by autoclaving.

**Table 1:** Bacterial strains und plasmids.

Strain	Genotype	Source/Ref.	
*E*. *coli* TOP10	F- *mcrA*, Δ(*mrr*-*hsdRMS*-*mcrBC*), φ80*lacZΔM15*, Δ*lacX74*, *nupG*, *recA1*, *araD139*, Δ(*ara*-*leu*)7697, *galE15*, *galK16*, *rpsL*(Str^R^), *endA1*, λ^−^	Invitrogen	
*E*. *coli* S17-1	Sm^R^, Tp^R^, *mod* ^+^, *res* ^−^, thi, pro, *recA* ^−^, RP4-Tc::Mu-Km::Tn7	Lab strain collection^49^	
*B*. *licheniformis* MW3	*B*. *licheniformis* DSM13 (*ΔhsdR1*, Δ*mcrA*, *ΔhsdR2*)	Prof. Dr. Friedhelm Meinhardt, University Münster^48^	
*B*. *licheniformis* MW3 AprAs^−^	*B*. *licheniformis* DSM13 (*ΔhsdR1*, Δ*mcrA*, *ΔhsdR2*, AprAs^−^)	this study	
*B*. *licheniformis* MW3 pV2::*aprAs*	*B*. *licheniformis* MW3 with pV2::*aprAs*	this study	
*B*. *licheniformis* MW3 AprA^−^ pV2::*aprAs*	*B*. *licheniformis* MW3 AprA^−^ with pV2::*aprAs*	this study	
*B*. *licheniformis* MW3 pV2	*B*. *licheniformis* MW3 with pV2	this study	
*B*. *licheniformis* MW3 AprA^−^ pV2	*B*. *licheniformis* MW3 AprA^−^ with pV2	this study	
**Plasmid**	**Genotype**	**Host**	**Source/Ref**.
pKVM2	*bla tet* ^*R*^ (*PclpB*-*bgaB*) oriT	*E*. *coli*, *Bacillus*	47
pKVM2 AprAs^−^	AprAs deletion vector	*E*. *coli*, *Bacillus*	this study
pV2	*bla*, *kan*, oriT	*E*. *coli*, *Bacillus*	33
pV2::*aprAs*	*bla*, *kan*, oriT, *aprAs*	*E*. *coli*, *Bacillus*	this study

### PCR, gel electrophoresis and vector construction

Primers used in this study are listed in Table 2 and plasmids in Table 1. PCRs (50 μl) consisted of 200 μM deoxynucleotides, 100 ng of template DNA, 5 pmol of each primer and 0.5 U Phusion High-Fidelity DNA Polymerase (Thermo Scientific, Darmstadt, Germany). PCR products were purified directly using the PCR Purification Kit (Qiagen, Hilden, Germany) or after gel electrophoresis using a Qiaquick Gel extraction Kit (Qiagen, Hilden, Germany). DNA was analysed using a TAE agarose gel system as described elsewhere^42^ and stained with Ethidium bromide (1 µg/mL) for 10 min. Vectors were constructed as described previously^30^ using *Escherichia coli* TOP10 (Invitrogen, Carlsbad, USA) and isolated using the QIAprep Spin Miniprep Kit (Qiagen, Hilden, Germany). For sequence verification Sanger-sequencing was performed on an ABI3730XL capillary sequencer (BigDye 3.1 chemistry; Applied Biosystems, Darmstadt, Germany).

**Table 2:** Primers.

Name	Sequence
HLRH300	*AAA* **GGATCCA**TTGGGCGGAGCAGACGTCAAAGCG
HLRH301	CTCAATAAC**GGCGCGCCA**CAAATGGCATATAGAAAAAGCTAGTGTTTTTAGCAC
HLRH302	GCCATTTGT**GGCGCGCC**GTTATTGAGCGGCAGCTTCGACATTG
HLRH303	*TTTT* **CCATGG**CTCGTGTTCACGATGGCCTTCAGC
HLRH304	GGCCGATCAGGCAGATATTC
HLRH305	GCATGTTGCCGGTACAGTAG
HLRH306	GGGAGCCTTTCTCCTGTATGTG
HLRH307	TCTTTCCCTGCCAGGTTGAAGC
HLRH308	TTGAGCGGCAGCTTCGACATTG
HLRH309	*CTAATACGACTCACTATAGGGAGA*CGGGAGCAGCAGCTTTGATCTTG
apr fwd	CACTAGCTTTTTCTATATGCCATTTG
apr rev	ACGGCGACTTATTTGGGAAGC
HLRH320	*CTGA* ***CGGCGCGCC***GACCATCAGGCTCAACAGCG
HLRH321	*TGCCCC* ***ACGCGT***GGATTGTGCTCTGGGATACCAC

Primer extensions are presented in italics and restriction sites in bold letters. The T7 RNA polymerase promoter is underlined and presented in italics.

### Mutant construction

For construction of *B*. *licheniformis* MW3 AprAs^−^, the markerless deletion protocol of Rachinger *et al*.^47^ was applied to the strain *B*. *licheniformis* MW3^48^, a restriction-modification (RM) system-negative derivative of *B*. *licheniformis* DSM13^23, 24^. The deletion cassette consisted of flank A (primer pair HLRH300/301) and flank B (primer pair HLRH302/303), which were amplified from *B*. *licheniformis* MW3 chromosomal DNA. The used primers included the *Asc*I restriction site instead of the −10 box of the putative AprAs promoter. The flanks were fused via SOE-PCR and cloned into the temperature-sensitive vector pKVM2 using *Bam*HI and *Nco*I restriction sites (*Bam*HI, *Nco*I, FastAP and T4-DNA-ligase (Thermo Scientific, Darmstadt, Germany)). *Escherichia coli* S17-1^49^ was used for conjugative transfer of the resulting pKVM2 AprAs^−^ deletion vector. The introduced deletion was confirmed by restriction analysis. For this purpose, the region of interest was amplified via PCR using primer HLRH306/307 and digested with the endonuclease *Asc*I. The introduced *Asc*I restriction site in the mutant strains results in the degradation of the 2.6 kbp PCR product to 1.3 kbp. The genotype of *B*. *licheniformis* MW3 AprAs^−^ was also confirmed by sequencing using primer pair HLRH306/307 and the primers HLRH304/305/306/307.

The complementation vector pV2::*aprAs* was constructed by amplification of *aprAs* from *B*. *licheniformis* MW3 chromosomal DNA using primers HLRH320/321 and cloning of the PCR product into the *Bacillus*/*E*. *coli* shuttle vector pV2^33^ using *Asc*I and *Mlu*I restriction sites.

For construction of the *B*. *licheniformis* plasmid harbouring strains, pV2::*aprAs* or the empty pV2 were transferred via conjugation using *Escherichia coli* S17-1^49^. The generated strains were controlled via plasmid re-isolation and restriction analysis.

### Slot Blot analysis

Cells were collected at different time points and disrupted with a Mikrodismembrator U (B. Braun Biotech International GmbH, Melsungen, Germany). Total RNA was prepared using the RNeasy Mini Kit (Quiagen, Hilden, Germany). The RNA quality was analysed using the Agilent 2100 Bioanalyser and the Agilent RNA 6000 Nano ladder (Agilent Technologies, Waldbronn). Digoxigenin labelled RNA probes were prepared by *in vitro* transcription with T7 RNA polymerase using the DIG Northern Starter Kit (Roche, Basel, Switzerland) and templates for *in vitro* transcription were generated by PCR using the primer pair HLRH308/309. Total RNA was diluted in 10x SSC to a concentration of 0.5 μg/100 μl and blotted on to a positively charged Nylon membrane (Roche, Basel, Switzerland) using the Bio-Dot SF microfiltration unit (BIO-RAD Laboratories GmbH, Munich, Germany). Subsequently, the RNA was covalently bound by exposing the membrane to UV-light (302 nm) for 120 sec on a UV-light table (Image Quant 100, GE Healthcare, Little Chalfont, UK). RNA probe hybridization was performed using the DIG Northern Starter Kit (Roche, Basel, Switzerland) following the manufacturer’s instructions and detection was accomplished via the ChemoCam Imager (Intes, Göttingen, Germany).

### Real-time PCR

Reverse transcription of 100 ng total RNA from each sample was performed using the RevertAid First Strand cDNA Synthesis Kit (Thermo Scientific, Darmstadt, Germany). For quantification of the *apr* cDNA the Real-time PCR Thermal Cycler IQ5 (Biorad, Munich, Germany) was used in combination with the QuaniTect SYBR® Green PCR Kit (Qiagen, Hilden, Germany) and the *apr* specific primer pair apr fwd/apr rev. For absolute quantification of *apr* transcripts a standard curve was calculated using vector pV2::*aprAs* with concentrations of 10^1^ to 10^8^ copies/µl.

### Protease assay

For exoprotease activity determination cells were cultivated 12–48 h in M9 skim milk medium and pelleted for 2 min at 16,200× g at 4 °C. The supernatants containing the exoprotease were analysed with Sigmas’ non-specific protease assay^50^. The protease activity for each sample was determined in triplicates using 2 mL Eppendorf tubes. Each sample contained one blank as reference.

### Preparation of extracellular protein extracts for 2D-PAGE

*B*. *licheniformis* cells were grown in M9 skim milk medium for 48 h and supernatants were harvested by centrifugation of 360 mL cell culture at 10,000 rpm and 4 °C for 10 min. Extracellular proteins were prepared according to Voigt *et al*.^51^. Briefly, TCA was added to the supernatant to a final concentration of 10% of the initial culture volume and proteins were precipitated at 7 °C overnight. The proteins were collected by centrifugation at 10,000 rpm and 4 °C for 60 min and washed 8 times with 96% ethanol, and two times with 70% ethanol. After drying, the pellets were dissolved in a solution containing 8 M urea and 2 M thiourea, and centrifuged at 15,000 rpm and 4 °C for 30 min. The protein concentration of the samples was determined with RotiNanoquant (Carl Roth, Karlsruhe, Germany).

### 2D-PAGE, gel image analysis and protein identification

Commercially available IPG strips (18 cm long, Serva, Heidelberg, Germany) in the pH-range 3–10 were used for isoelectric focusing (IEF). 100 µg protein was adjusted to 306 μL with a solution containing 2 M thiourea and 8 M urea. CHAPS solution (20 mM DTT, 1% w/v CHAPS, 0.5% v/v Pharmalyte, pH 3–10) was added (34 µL for each sample). IEF strips were rehydrated with this solution over night. IEF was performed with the following program: step 1, 150 V for 150 Vh, step 2, 300 V for 300 Vh, step 3, 600 V for 600 Vh, step 4, 1500 V for 1500 Vh, step 5, 3000 V for 37.5 kVh. Following IEF, strips were equilibrated as described by Görg *et al*.^52^. Flat top gels (2D HPE^TM^ Large Gel NF-12.5%; Serva, Heidelberg, Germany) were used for protein separation in the second dimension. Gels were stained with Lava Purple (Fluorotechnics, Sydney, Australia) according to the instructions of the manufacturer. Gel images were analyzed with the Delta2D software (Decodon, Greifswald, Germany). Spot quantification was done according to Wolf *et al*.^34^. Briefly, gel images (three replicates) from the wild type samples were overlaid with the gels from the mutant samples. Fusion gels created with the image fusion function of the Delta2D software set to union fusion were used for spot detection. Spots were edited and transferred to the single gels. For spot quantification the % volume of each spot was calculated representing the relative portion of an individual spot of the total protein present on the gel.

Proteins were excised from the gels using the Ettan Spot Picker (GE Healthcare, Little Chalfont, UK). Digestion and spotting onto MALDI targets were performed in the Ettan Spot Handling Workstation (GE Healthcare, Little Chalfont, UK). MALDI-TOF-MS/MS with the Proteome Analyzer 4800 (Applied Biosystems, Darmstadt, Germany) was performed as described by Wolf *et al*.^34^. Peak lists were searched with the MASCOT search engine version 2.1.0.4 (Matrix Science, London, UK) and search parameters as in Wolf *et al*.^34^ against a *B*. *subtilis* data base.

### Sequence analysis

For identification of the putative *aprAs* promoter region in 17 *B*. *licheniformis* strains and *B*. *subtilis* 168, the intergenic region between *yhfN* and *apr* from *B licheniformis* DSM13 was used to perform a blastn search. Correspondingly, the intergenic region between *yhfN* and *aprE* from *B*. *subtilis 168* was used as query for a blastn search on 35 *B*. *subtilis* genomes. The identified regions were extracted from the respective genomes and used for a comparative sequence alignment performed with MUSCLE^53^ using default parameters.

Transcriptional terminators were predicted with the program TransTermHP^54^. Secondary structure predictions were performed using “The Vienna RNA Websuite”^55^.

## Electronic supplementary material

Supplementary information
