# Supplementary material for: Small RNA mediated repression of subtilisin production in Bacillus licheniformis
Source: Sci Rep. 2017 Jul 18;7:5699. doi: 10.1038/s41598-017-05628-y (PMC5516005; doi:10.1038/s41598-017-05628-y)
Supplement: Supplementary file 1 — Supplementary information [file 41598_2017_5628_MOESM1_ESM.pdf]

Small RNA mediated repression of subtilisin production in *Bacillus licheniformis*

Robert Hertel, Sandra Meyerjürgens, Birgit Voigt, Heiko Liesegang and Sonja Volland

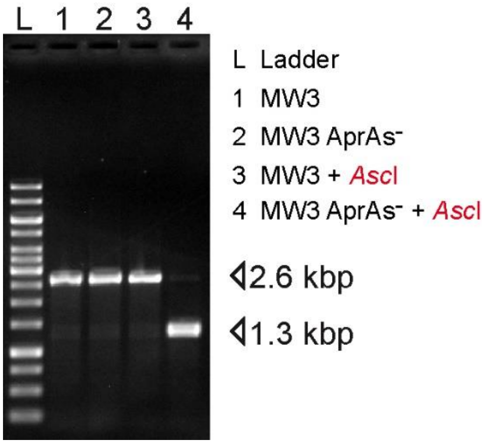

Supplementary Figure S1: Detection of *B. licheniformis* MW3 AprAs- mutant strains. PCR products, amplified from original (MW3) and mutant cells (MW3 AprAs-), were digested with the endonuclease *Asc*I and separated by gelelectrophoresis. The PCR product from the desired mutant *B. licheniformis* AprAs- exhibited an expected 1.3 kbp DNA band consisting of the degradation products of the 2.6 kbp PCR product (lane 4). The PCR products without *Asc*I treatment (lane 1 and 2) as well as the *Asc*I treated PCR product from *B. licheniformis* MW3 exhibited a band in the size of the 2.6 kbp PCR product.

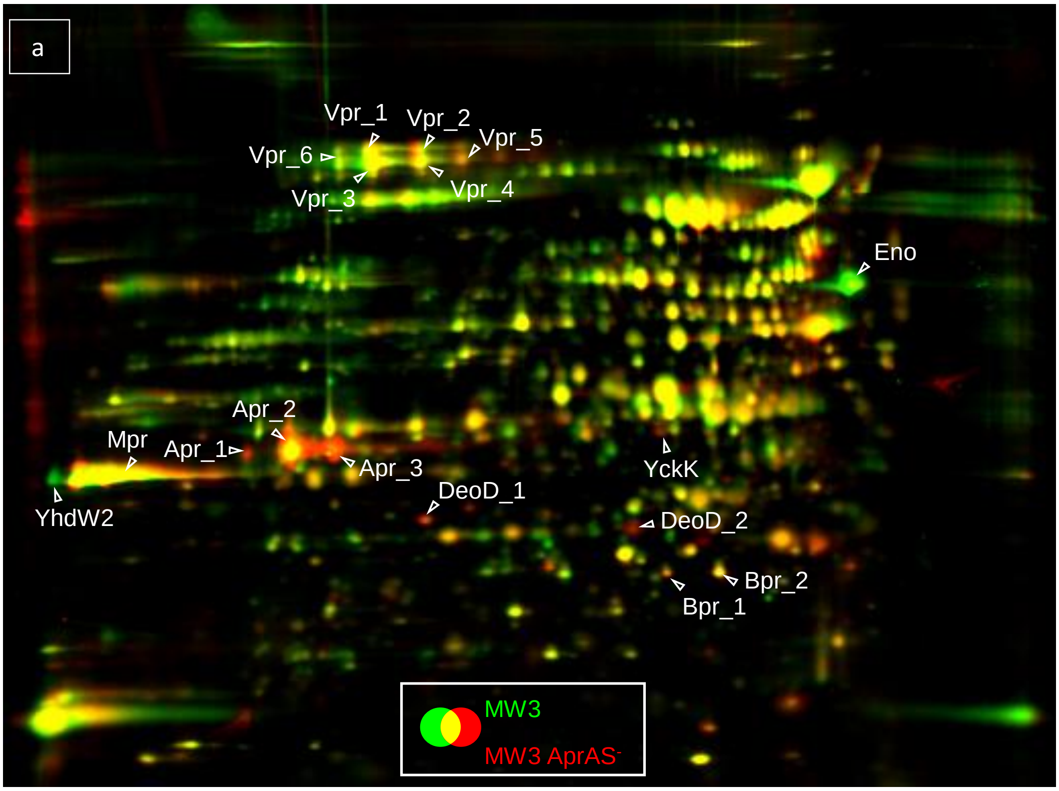
a
Vpr_1
Vpr_2
Vpr_5
Vpr_6
Vpr_3
Vpr_4
Eno
Apr_2
Mpr
Apr_1
Apr_3
YckK
DeoD_1
YhdW2
DeoD_2
Bpr_2
Bpr_1
MW3
MW3 AprAS-

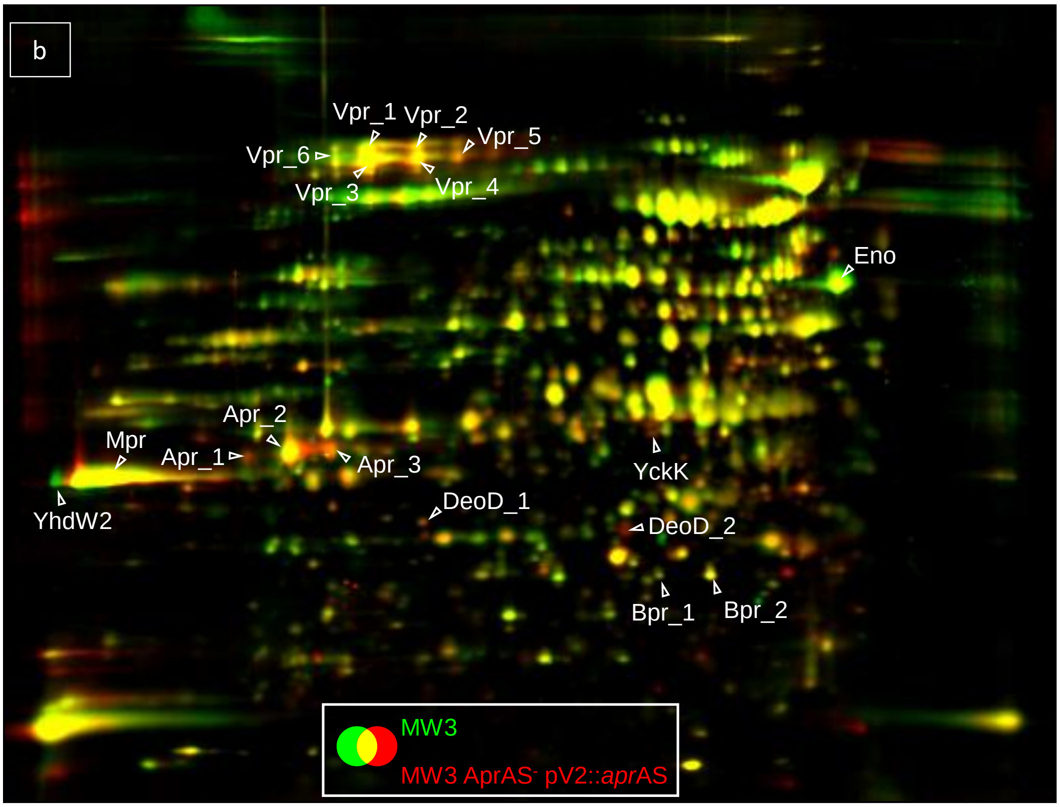
b
Vpr_1
Vpr_2
Vpr_5
Vpr_6
Vpr_3
Vpr_4
Eno
Apr_2
Mpr
Apr_1
Apr_3
YckK
DeoD_1
YhdW2
DeoD_2
Bpr_1
Bpr_2
MW3
MW3 AprAS- pV2::aprAS

Supplementary Figure S2: Extracellular proteome of *B. licheniformis* MW3, MW3 AprAS$^-$ and MW3 AprAS$^-$ pV2::*aprAs* analysed by 2D-gelelectrophoresis. Extracellular proteins were isolated from supernatants of 400 ml M9 skim milk cultures after 48h of growth. The protein fractions were separated by 2D-gelelectrophoresis and are shown as dual channel images. **a.** A Protein blot from *B. licheniformis* MW3 (green) was overlaid with the respective blot from MW3 AprAs$^-$. **b.** A Protein blot from *B. licheniformis* MW3 (green) was overlaid with the respective blot from MW3 AprAs$^-$ pV2::aprAs (red). Protein spots of the other known extracellular *B. licheniformis* proteases*,* like the metalloprotease Mpr and the minor extracellular protease Vpr (Vpr_1 – Vpr_6), as well as the extracellular bacillopeptidase Bpr (Bpr_1, Bpr_2) are not differentially expressed. The amount of the enolase Eno and the putative glycerophosphoryl diester phosphodiesterase yhdW2 is decreased and the purine nucleoside phosphorylase DeoD and YckK is increased in *B. licheniformis* MW3 AprAs- compared to the original strain *B. licheniformis* MW3 (a). The observed differential expression of these proteins is still visible after complementation with the vector pV2::*aprAs* (b).

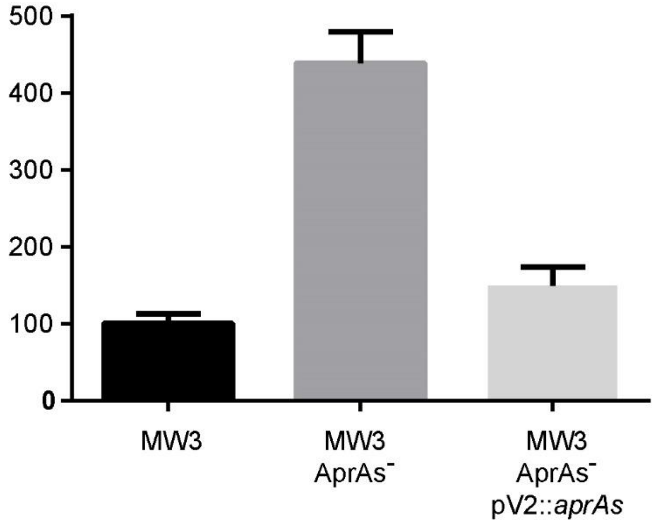

Supplementary Figure S3: Comparison of *apr* transcript levels of *B. licheniformis* MW3, MW3 AprAs⁻ and MW3 AprAs⁻ pV2::*aprAs*. The total RNA of samples taken at 48h of growth was reverse transcribed and the *apr* mRNA transcript level was evaluated by Real-time PCR analysis using *apr*-specific primers. The *apr* transcript number of *B. licheniformis* MW3 was set 100 and the standard deviation was given for two independent analyses with 3 replicates each. The actual *apr* transcript numbers of 5 ng total RNA are shown in Supplementary Table S1. The *apr* transcript level after silencing of AprAs (see *B. licheniformis* MW3 AprAs⁻) is approximately 4 times increased compared to *B. licheniformis* MW3. After complementation with vector-encoded *aprAs* (see *B. licheniformis* MW3 AprAs⁻ pV2::*aprAs)* the *apr* transcript level is again decreased to approximately 1.5 times of the level of the initial strain MW3.

Supplementary Table S1: *apr* transcript numbers of *B. licheniformis* MW3, MW3 AprAs⁻ and MW3 AprAs⁻ pV2::*aprAs* determined by Real-time PCR

**Sample 1**	***apr* transcripts**	**Sample 2**	***apr* transcripts**
MW3_1.1	287000	MW3_2.1	850000
MW3_1.2	265000	MW3_2.2	737000
MW3_1.3	248000	MW3_2.3	1060000
MW3_aprAs⁻_1.1	1280000	MW3_aprAs⁻_2.1	3680000
MW3_aprAs⁻_1.2	1130000	MW3_aprAs⁻_2.2	3350000
MW3_aprAs⁻_1.3	1310000	MW3_aprAs⁻_2.3	3870000
MW3_aprAs⁻_pV2::aprAs_1.1	466000	MW3_aprAs⁻_pV2::aprAs_2.1	1200000
MW3_aprAs⁻_pV2::aprAs_1.2	463000	MW3_aprAs⁻_pV2::aprAs_2.2	1030000
MW3_aprAs⁻_pV2::aprAs_1.3	435000	MW3_aprAs⁻_pV2::aprAs_2.3	1160000

The *apr* transcript numbers were determined from 5 ng total RNA.
